# Effect of *Onopordon acanthium* L. as Add on Antihypertensive Therapy in Patients with Primary Hypertension Taking Losartan: a Pilot Study

**DOI:** 10.15171/apb.2018.009

**Published:** 2018-03-18

**Authors:** Roshanak Ghods, Manouchehr Gharouni, Massoud Amanlou, Niusha Sharifi, Ali Ghobadi, Gholamreza Amin

**Affiliations:** ^1^Research Institute for Islamic and Complementary Medicine, Iran University of Medical Sciences, Tehran, Iran.; ^2^School of Persian Medicine, Iran University of Medical Sciences, Tehran, Iran.; ^3^Faculty of Medicine, Tehran University of Medical Sciences, Tehran, Iran.; ^4^Department of Medicinal Chemistry, Faculty of Pharmacy, Tehran University of Medical Sciences, Tehran, Iran.; ^5^Department of Pharmacognosy, Faculty of Pharmacy, Tehran University of Medical Sciences, Tehran, Iran.

**Keywords:** Onopordon acanthium L, Hypertension, Blood pressure, Angiotensin converting enzyme, Persian medicine

## Abstract

***Purpose:*** Onopordon acanthium L. is known for its medicinal properties. Our recent study showed that its seed extract is a novel natura angiotensin-converting-enzyme inhibitor (ACEI). This study was carried out to investigate its possible antihypertensive effects in patients receiving losartan.

***Methods:*** This uncontrolled clinical trial was carried out among 20 patients (30-60y) with uncontrolled hypertension despite receiving 50 mg losartan (stage I & II) in two hospitals in Iran. After completing informed consent, patients were treated by 2 capsules [each 1g of Onopordon acanthium seed extract (OSE)] as add-on therapy, two times per day.

***Results:*** 18 patients completed the study (50.94 ±8.37y). Mean systolic blood pressure (SBP) at the baseline was 151.9 ± 13.74mmHg and at the end of the study, it was 134.6 ± 18.25 mmHg and mean diastolic blood pressure (DBP) was 97.41 ± 10.36 at the baseline and was 85.71 ± 7.481 after 8 weeks. OSE significantly reduced SBP and DBP at the end of 8 weeks (P=0.003, 95% CI: -19.7, -15.1; P=0.0006, 95% CI: -10.23, -13.15; respectively). No evidence of hepatic or renal toxicity was detected.

***Conclusion:*** Based on the results of this study OSE has antihypertensive property with no significant adverse effects. However, because of the low number of samples, this medication may be not safely administered. The results of this study could be the basis for further studies with larger sample size. IRCT registration number: IRCT2013020712391N.

## Introduction

Hypertension (HTN) is a global concern and affects approximately 75 million adults in the United States and if left untreated increases risk of stroke, myocardial infarction, vascular disease, and chronic kidney disease.^1,2^ Diagnosis and treatment of high blood pressure are essential to prevent mortality and morbidity.^2,3^ Hypertension may be treated by using routine drugs such as angiotensin-converting-enzyme (ACE) inhibitors, beta-blockers, diuretics, calcium channel blockers, alpha-blockers, and peripheral vasodilators,^3,4^ improving lifestyle factors including weight loss, quitting smoking, reducing sodium intake, regular exercise and limiting alcohol consumption.^5^ These recommendations may be used alone or in combination with others^4^. Losartan is an oral medication that belongs to a class of drugs called angiotensin receptor blocker (ARBs) which was approved by the U.S. Food and Drug Administration (FDA) in April 1995. Losartan blocks the angiotensin receptor, relaxes muscle cells and dilates blood vessels and reduces blood pressure.^6,7^

Our recent study showed that the OSE is a novel natural inhibitor agent and inhibit angiotensin-converting-enzyme by 80.2 ± 2 % at‏ concentration‏ of‏ 330 μg/ml, and exerted‏ antioxidant‏ activity (IC_50_ value‏ of‏ 2.6 ± 0.04 mg/ml).^8^ This plant has been well known under the name "Khaje Bashi"^9^ and has long been used in folk medicine as a hypotensive, cardiotonic and diuretic agent.^10^ Also it has been noted in Persian Medicine (PM) literatures as diuretic, diaphoretic, antipyretic, analgesic. Cotton thistle or Scotch thistle are its common names. It is a flowering plant belonging to the Compositae (Asteraceae) family and is widely naturalized almost globally, particularly in Europe and Western Asia and is a vigorous biennial plant with coarse, spiny leaves and conspicuous spiny-winged stems.^11^ In modern medicine, *O. acanthium* has been reported to be a bactericide, cardiotonic, hypotensive and hemostatic agent and is used against hypotonicity.^12-14^ This species has several bioactive components among which sesquiterpene lactones have been found to have numerous‏ biological properties including antibacterial, anti-inflammatory, anti-malarial, and hypotensive effects‏.^15^ Since the antihypertensive effect of *O. acanthium* seed extract has not been studied on patients in follow up of our previous in vitro study, we conducted this trial to investigate the possible antihypertensive effects of OSE on patients with stage I-II hypertension who were under treatment with losartan, a chemical ACE inhibitor, as add-on therapy.

## Materials and Methods

### Plant material

*O. acanthium* L. dried seeds were purchased from the local market in Tehran (Grand Bazaar), Iran. The seeds were identified by Prof. Gholamreza Amin and were kept under the voucher number PMP–714 at the herbarium of Faculty of Pharmacy, Tehran University of Medical Sciences.

### Preparation of the extract and drug formulation

The extract was prepared in the laboratory of traditional pharmacy at faculty of Traditional Medicine, Tehran university of medical sciences via maceration method using ethanol as the solvent (1:8) at three time points (24, 48 and 72 h). The three extracts were mixed, filtered and evaporated. The total evaporated extract was freeze-dried and grinderies to obtain a powder for preparing the capsule. Each capsule contained 1g of the freeze-dried powder equal to 11 gram of dried seeds, and all the physiochemical quality control (QC) tests were performed on the capsules.

### Toxicology evaluation

To measure the toxic dose of total extract of OSE (*Khaje Bashi* plant), rats were used and kept at the Animal house of the Faculty of Pharmacy, Tehran University of Medical Sciences. Animals were kept in standard light (12 hours of light and 12 hours of darkness), room temperature (25-35 °C) with adequate water and food. 12 male albino NMRI mice were divided into three groups (4 animals per group). The whole extract of the plant was taken in dried form and stored in a dark bottle in the refrigerator until it was tested. Due to its low solubility, the extract was dissolved in dimethyl sulfoxide (DMSO) at a desired concentration and diluted with normal saline at a rate of 1:3 and made as a monotonous suspension.

The solution was injected intraperitoneally (i.p.). Animals received the same amount of DMSO in different dose groups. Animals’ condition (lethargy and movement) and animal death were investigated for 24-48 hours. In each group, mortality rate was reported and plotted against the dose used. The dose that causes 50 percent of deaths in animals or “Lethal Dose, 50%” (LD_50_) was calculated based on the curve.

Considering that no studies have been done on the toxicity of this plant, in the first step, 0.5, 1, 2, 3.5 and 5 g/kg of body weight were used to find the dose range, but no mortality or change was observed in animals. In the next step, doses of 6-20 g/kg were used, and in dose 20, all animals died. In the next stage, the range of 6-13.5 g/kg was tested in 6 groups of animals. The results were calculated as a percentage of death for each group. LD_50_ was calculated using non-linear sigmoid regression and Probit method. Meanwhile, the relationship between effective dose and effect was analyzed using Pearson's relationship and according to the results of these experiments, the lethal dose (LD_50_ value of 8.44 ± 0.04 g/kg) was determined. According to these results, the extract of this plant was classified in "practically non-poisonous ".

### Clinical trial design

This open-labeled, non-randomized, uncontrolled clinical trial, a pilot study, was performed on 20 patients, who were under losartan treatment (50mg/d) for at least 6 weeks before starting the study and their blood pressure constantly remained higher than 140/90 (stage I&II) hypertension according to the seventh report of the joint national committee (JNC VII) report‏.^16^ Sample size was calculated as follow: based on predicted 14 mmHg reduction of systolic blood pressure (d=14) at the end point (after 8 weeks), standard deviation 20 (σ=20, power 80%, confidence interval (CI) of 95% (Z_1-α/2_=1.96) and taking into account a correlation of 0.5 between frequent measurements and considering 20 % loss to follow, 20 patients were enrolled to determine the effective dose of therapy. Each individual took 2 capsules, two times a day‏ (2 g/BD) and blood pressure was checked every other week. If blood pressure has increased more than 15 mmHg, the patients would have dropped out the study.

The primary outcome measure was systolic and diastolic blood pressure that was measured using aneroid sphygmomanometer (F. Bosch, Model:‏ 0123, Germany). Metabolic parameters (lipid profile, liver function tests, BUN, Cr, FBS) were measured two times during the study (at the beginning and at the end of study) at Noor laboratory to evaluate the general health status of the patients.

### Participants

The patients (30-60 y) were selected from Imam Khomeini & Amir-alam hospitals of Tehran University of medical sciences. Patients with history of malignant hypertension, blood pressure higher than 200/140 mmHg and whom needed multi-drug treatment or emergency IV drug infusion, history of secondary hypertension, end organ damages (EOD), sudden increase of blood pressure to greater than 15 mmHg at any time during the study, cardiac arrhythmias, symptomatic valvular heart diseases (except mitral valve prolapse) were excluded from the study. Other exclusion criteria were: diabetes type 1& 2, liver disorders, pregnancy breast feeding, cured or uncured malignancies during the last 5 years, serum potassium >5.2 or <3.5 mEq/L in the first visit, drug or alcohol abuse. The procedure was explained to all patients and written informed consents were obtained. Patients were examined at the beginning of the study and then every two weeks, and their arterial blood pressure was measured. The researcher's contact number was also provided to patients in order to report any problems between every two visits. In addition, patients were asked at each session about possible complications such as coughing, severe headache, visual impairment, any types of arrhythmias, and orthostatic hypotension, and were recorded. Demographic and baseline data, medical history and any concomitant medications of enrolled patients were recorded. The study protocol was approved by the ethics committee of Research Institute for Islamic and Complementary Medicine of Iran University of Medical Sciences at 09/10/2012 with reference number 732/P26/M/T.

### Statistical methods

The changes in blood pressure were analyzed using repeated measures ANOVA analysis. P value <0.05 was considered significant. The LD_50_ was calculated using nonlinear sigmoid regression and Probit technique. Moreover, the correlation between effect and administered dose was analyzed using Pearson correlation test.

## Results and Discussion

**Figure 1:**
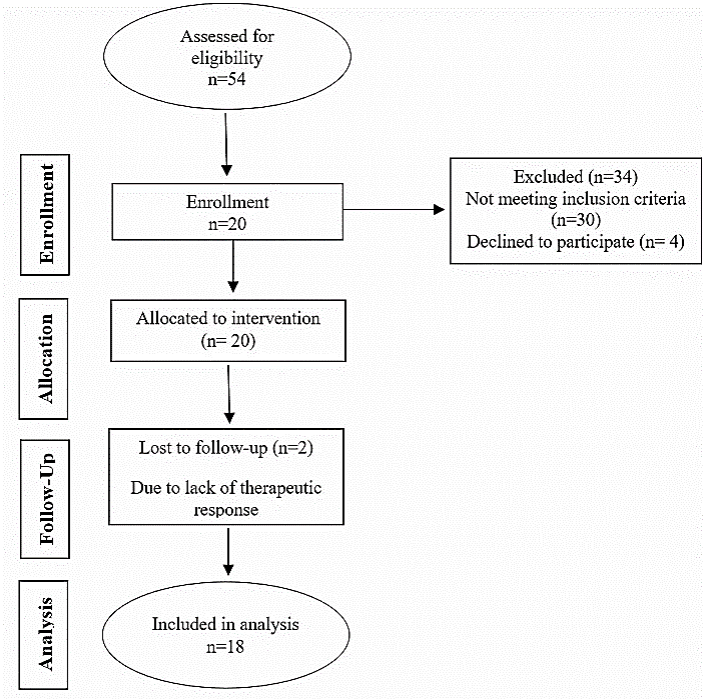
The trial flowchart

Cytotoxicity of the extract was performed in mice by injecting different doses of OSE (0.5-13.5 g/kg) to measure the minimum toxic dose of OSE and LD_50_ was 8.44 ± 0.04 g/kg. Sampling began on May 21, 2013 and continued for five months, till October 23, 2013. 54 patients were assessed. Only 24 patients met the inclusion criteria whom four were not willing to participate in the study. At the end, 18 patients (of 20) completed the study and two patients were left the study due to increased blood pressure more than 160/100 mmHg in one patient after 2 days and another, after 3 days from starting point. Based on the ethical issues and respect to the patients’ right, they were excluded and researcher referred both of them to the cardiologist to be treated with two or more drugs (Figure 1).

Patients took 2 capsules two times a day (2 g/BD) and were visited every other week and systolic and diastolic blood pressure and any possible side effects were recorded (Table 1).

**Table 1:** Blood pressure changes (before and after the study)

**Blood pressure(mmHg)**	**Time**	**Mean ± SD**
**Systolic blood pressure (SBP)**	at baseline	151.9 ± 13.74
	after 8 weeks	134.6 ± 18.25
**Diastolic blood pressure (DBP)**	at baseline	97.41 ± 10.36
	after 8 weeks	85.71 ± 7.48

**Figure 2:**
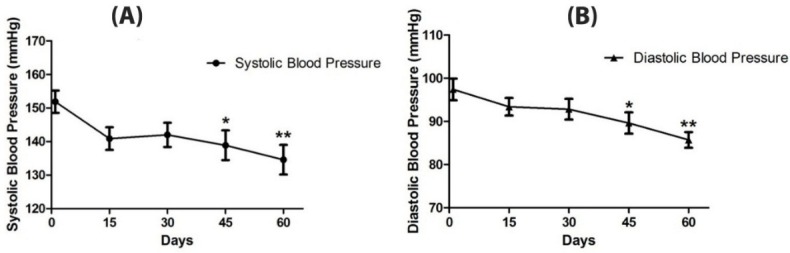
Blood pressure changes during treatment**.** Systolic (A) and diastolic blood pressures (B) have been measured at multiple time points: on the first day of investigation, 15, 30, 45, and 60 days after treatment (mean ± SEM). Decline of blood pressure at different time points have been compared with the blood pressure at the beginning of the investigation. There was a significant decline of blood pressure beyond 45 days of treatment when compared with the blood pressure before treatment (the asterisks indicate P < 0.05, repeated measures ANOVA analysis).

After treatment for at least 8 weeks, systolic and diastolic blood pressure decreased significantly (P=0.003, 95% CI: -19.7, -15.1; P=0.0006, 95% CI: -10.23, -13.15; respectively) (Table 2). Moreover systolic and diastolic blood pressure decreased after 45 days treatment (P=0.025, 95% CI: -15.4, -10.6; P=0.034, 95% CI: -7.71, -7.85; respectively). At the end of the study, OSE decreased systolic and diastolic blood pressure 17.3 and 11.7 mmHg, respectively. (Figure 2, A & B).

**Table 2:** Baseline characteristics of participants and changes in systolic and diastolic BP.

**Demographic characteristics**		
**Age**	y (mean ± SD)	50.94 ± 8.73
**BMI**	kg/m^2^(mean ± SD)	30.4 ± 4.84
**Male**	n (%)	7 ( 35)
**Sample size**	n	20
**Blood pressure(mmHg)**		
**Systolic blood pressure (SBP)**	After 2 weeks	~ -11
	After 4 weeks	~ -9.9
	After 6 weeks	~ -13
	After 8 weeks	~ -17.3
**Diastolic blood pressure** **(DBP)**	After 2 weeks	~ -4.3
	After 4 weeks	~ -4.59
	After 6 weeks	~ -7.79
	After 8weeks	~ -11.7

We did not observe hepatic or renal toxicity as the result of *O. acanthium* extract consumption in patients during the study‏.

**Figure 3:**
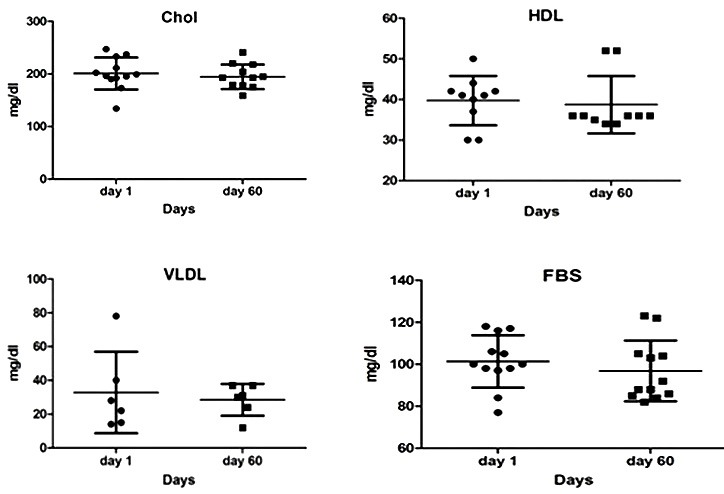
Changes in metabolic factors compared with the beginning of the intervention. After analysis, student t-test did not change significantly. P <0.05 was considered as a significant level.

**Figure 4:**
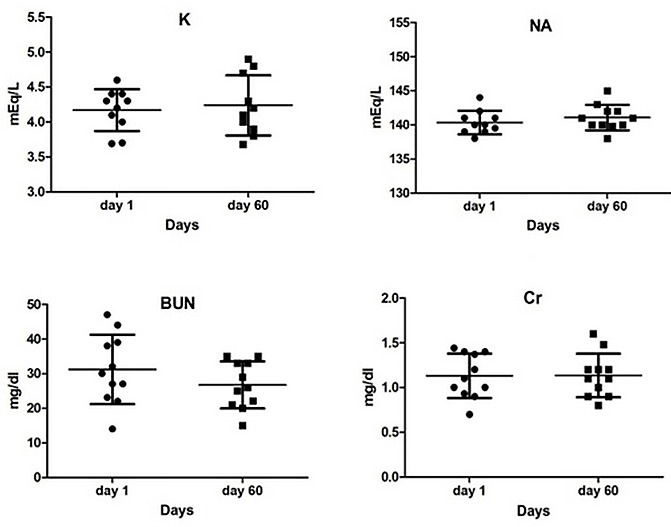
Changes in electrolytes and renal tests compared with the beginning of the intervention. After analysis, student t-test did not change significantly. P <0.05 was considered as a significant level.

**Figure 5:**
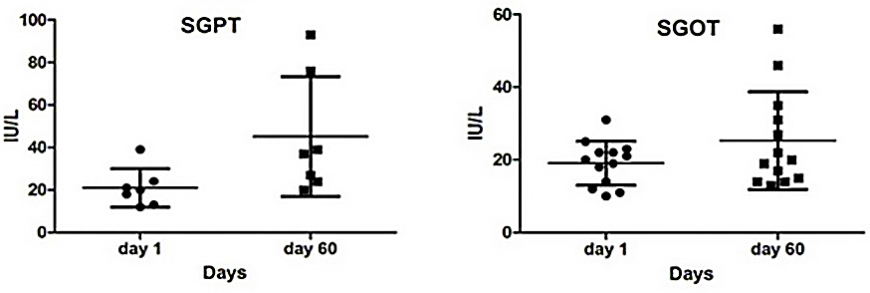
Changes in the liver function tests compared to the beginning of the intervention. After analysis, student t-test did not change significantly. P <0.05 was considered as a significant level.

The results of these tests showed no significant changes (CBC, FBS, TG, Chol, LDL, HDL, SGOT, SGPT, BUN, Cr). As shown in the following diagrams, although these changes were not significant, in some cases, such as glucose, cholesterol, triglyceride, LDL, VLDL, and BUN, there was a tendency to reduction. Based on these results, HDL levels show a modest increase. The levels of sodium and creatinine were almost constant. The amount of potassium and liver enzymes were in the normal range (Figures 3 & 4). In general the changes of liver enzymes levels before and after treatment was not significant (P=0.1481; P=0.1400, respectively) (Figure 5).

Regarding the side effects, we detected mild dyspnea in two patients‏ after 2 weeks. Also two patients showed dizziness and feeling of heaviness in the head in the first month of treatment that resolved later.

These data indicated that 4 g/day of *O. acanthium* seed extract for 8 weeks significantly decreases systolic and diastolic blood pressure in patients with stage I and II primary essential hypertension under losartan treatment. In line with our recent findings in which a novel compound was extracted and identified from *O. acanthium* named Onopordia,‏ OSE showed antioxidant and angiotensin converting enzyme inhibitor (ACEI) activity (80.2 ± 2 %). Its potential of blood pressure decreasing can be due to its different components such as sesquiterpenes and flavonoid.^8^ There is no study on evaluating the effects of *O. acanthium* on blood pressure; however, some studies‏ around the world have evaluated the hypotensive effect of some popular and well-known hypotensive herbal medicines.

Asgary et al., in a study in Iran, administered *Achillea wilhelmsii* to 120 patients for 8 weeks and showed significant decline of systolic and diastolic blood pressure (P=0.005 and P=0.003, respectively)‏.^17^ Walker et al., administered 500 mg/day of dried, full-spectrum aqueous-alcoholic extract of hawthorn (*Crataegus laevigata*) leaves and flowers to 36 patients with essential HTN for 10 weeks and found that diastolic blood pressure decreased in 19 patients. However the difference was not significant (P=0.08)‏.^18^ In a follow up study Walker et al., evaluated the hypotensive effect of *Crataegus laevigata,* 1200mg/day for 16 weeks on 76 patients and indicated significant decrease in diastolic (P=0.03) but no significant decrease in systolic blood pressure (P=0.32)‏.^19^‏ Consistent with these findings Ried, et al. indicated that treatment with aged garlic extract for 12 weeks lowered systolic blood pressure 10.2 mmHg‏.^20^ Furthermore Susalit, et al., investigated the hypotensive effect of olive (*Olea europaea*) leaf extract, 500 mg/daily for 8 weeks on 232 patients and indicated the extract lowered 10 mmHg systolic blood pressure‏.^21^‏ Moreover, several other herbal agents were studied independently and showed hypotensive effect. Examples are Sour Cherry (*Prunus cerasus* L.), *Nigella sativa* seed extract, pomegranate juice, *Hibiscus sabdariffa* and canola oil with sunflower oil‏.^22-29^ In this study, OSE lowered systolic blood pressure by 17.3 mmHg that is more potent than all studied hypotensive agents. Similar effects have been reported about aged garlic extract and olive leaf extract that showed a reduction of about 10 mmHg of systolic blood pressure. The reasons for such discripency is not clear but it may be due to the presence of both ACEI and diuretic activity in OSE. This finding was in line with previous report by Sharifi, et al., that revealed OSE has more hypotensive effect than other 50 herbal agents which has been assessed‏.^8^

Several limitations are inherent to the present study. Recruiting hypertensive patients categorized in the first and second stage of hypertension who were also treated with losartan was very difficult. The majority of hypertensive patients had to use two or more drugs to control their blood pressure, due to lack of good response to one-drug therapy. The limitation of recruiting hypertensive patients in this study was consistent with the one in the other studies. The withdrawal of the patients was also an obstacle to obtain the desired results with higher power of analysis.

## Conclusion

OSE synergistically with diuretic and plasma ACE inhibitor activity reduced blood pressure (both systolic and diastolic) in the patients under treatment with losartan, and did not show remarkable side effects in patients with primary hypertension. Because of the low number of samples, this medication may be not safely recommended. Further clinical trials is needed for increasing certainty with larger sample size and placebo.

## Acknowledgments

This study was part of a postgraduate thesis entitled: ''Investigating the Effect of Onopordon Acanthium Seed on Primary Hypertension Reduction in Patients under Treatment by Losartan''; and was supported by a grant from Tehran University of Medical Sciences. The funding source had no involvement in any part of the study. We would like to thank the participants for their contribution to the maintenance of our patient record without which this project would have been impossible. Special thanks to Mr. Hadi Salehi, for his kindly efforts for extraction and drug preparation. We would like to thank Dr. Mohsen Amin assistant Prof. at Faculty of Pharmacy, Tehran University of Medical Sciences for his help in data analysis and manuscript editing.

## Ethical Issues

The study protocol was approved by the ethics committee of Research Institute for Islamic and Complementary Medicine of Iran University of Medical Sciences at 09/10/2012 with reference number 732/P26/M/T.

## Conflict of Interest

The authors have declared no conflicts of interest.
